# Primary Dengue and Long-Term Health Status in Madeira Island, Portugal: A Retrospective Questionnaire-Based Study

**DOI:** 10.4269/ajtmh.23-0502

**Published:** 2024-07-02

**Authors:** Paulo Henriques, Helena Caldeira-Araújo, Maria da Luz Brazão, Ana Maria Abreu, Ana Margarida Vigário, Alexandra Rosa

**Affiliations:** ^1^ Projeto Medicina, Faculdade de Ciências da Vida, Universidade da Madeira, Funchal, Portugal;; ^2^ CQM-Centro de Química da Madeira, Universidade da Madeira, Funchal, Portugal;; ^3^ Serviço de Medicina Interna, Hospital Central do Funchal, SESARAM-EPERAM, Funchal, Portugal;; ^4^ Departamento de Matemática, Faculdade de Ciências Exatas e da Engenharia, Universidade da Madeira, Funchal, Portugal;; ^5^ Centro de Investigação em Matemática e Aplicações, Universidade de Évora, Évora, Portugal;; ^6^ Instituto de Medicina Molecular João Lobo Antunes, Faculdade de Medicina, Universidade de Lisboa, Lisboa, Portugal

## Abstract

Dengue is among the most important mosquito-borne viral diseases worldwide. Although its acute manifestations are well known, little is known about the long-term impact of dengue on the population’s health status. Madeira Island experienced a single outbreak of autochthonous dengue from September 2012 to March 2013. To extend our knowledge about the clinical impact of the outbreak on this naive population, we applied an online questionnaire to 168 adults diagnosed with dengue at the time to characterize retrospectively their symptoms during the infection and to identify long-term manifestations, possibly triggered by dengue. The most frequent symptoms during the clinical period, reported by more than three-quarters of our participants, were fever, myalgia, extreme tiredness, and headaches, whereas vomiting, pruritus, nausea, retro-orbital pain, and arthralgia occurred in 35% to 50% of participants. In the 8 years after dengue, 61.5% of participants reported at least one recurrent previously nonexistent symptom, the most frequent being headaches, abundant hair loss, extreme tiredness, arthralgia, and myalgia, experienced by 25% to 35% of participants. Nearly 20% of the participants with persistent symptoms reported the onset of chronic illness in the 4 years after dengue, most frequently ophthalmological and autoimmune diseases (5.6% each), versus only 2.2% of chronic disease onset in participants without persistent symptoms. Our results suggest that the occurrence of persistent symptoms after primary dengue might be more frequent than anticipated and may persist for several years, having an impact on the health status and well-being of a considerable proportion of the infected population.

## INTRODUCTION

Dengue, a vector-borne disease caused by one of the four known serotypes of dengue virus (DENV), has become endemic in many tropical and subtropical regions,^1^ where it represents a major health concern. During the past 5 decades, the incidence of dengue epidemics has increased 30-fold, reaching nowadays 390 million annual infections, with new countries being affected.^2^

Infected people may be asymptomatic (estimated to be 50%–98% of the infected)^3^ or exhibit a wide spectrum of manifestations, from mild disease to severely debilitating or lethal disease, classified as “severe dengue” according to the WHO.^4^ Literature describing the manifestations of symptomatic dengue at the acute and convalescence phases of the infection is abundant.^5^^–^^8^ The most common signs and symptoms are fever accompanied by myalgia/arthralgia, headaches, retro-orbital pain, maculopapular rash, leukopenia, abdominal pain, and/or vomiting; in most patients, this is a self-limiting infection lasting up to 2 weeks.^1^^,^^4^^–^^8^

To gain a better understanding of the real impact of a dengue outbreak on the health status of a population, it is crucial to evaluate the clinical manifestations during active infection but also those that become more evident or appear after the recovery phase and persist chronically. Despite the recognition of late complications and unusual manifestations by the WHO,^9^ little is known about the long-term consequences of dengue. Some reports indicated that muscle pain, arthralgia, or asthenia, among other symptoms, may persist for 1–2 months in 9–24% of patients.^10^^–^^12^ A few studies have published evidence of an even longer duration of persistent symptoms, up to 2 years after infection.^6^^,^^13^^,^^14^ Additionally, long-term manifestations such as liver injury,^15^ cardiovascular sequelae (reviewed in Rahim et al.),^16^ and neurologic and ocular complications (reviewed in Trivedi and Chakravarty)^17^ have also been described. Infection with DENV can also favor Guillain–Barré or chronic fatigue syndromes,^17^^,^^18^ as observed in other viral infections.^19^^,^^20^

The only outbreak of autochthonous dengue in Madeira Island, Portugal, was caused by DENV1 serotype and occurred between September 2012 and March 2013, leading to more than 2,000 dengue cases assisted in public healthcare, without severe clinical forms or fatalities.^21^ The clinical picture when seeking medical assistance, which generally happened during the febrile period, may have been underestimated because some of the dengue manifestations only appear later on (e.g., petechiae) or may be temporary (e.g., rash).^22^ Additionally, many of the patients did not qualify for follow-up or further clinical surveillance, and thus no registry of expanded manifestations exists beyond the acute phase of the infection.

To deepen our knowledge about the clinical impact of the Madeira 2012 dengue outbreak, we applied an online questionnaire to dengue cases diagnosed at the time, aiming at 1) a retrospective characterization of their symptoms during the entire infection period, from the early onset of symptoms to the convalescence phase, and 2) an assessment of their health status before and after dengue, identifying long-term manifestations possibly triggered by dengue. We expected that the clinical manifestations reported here are a fair representation of DENV1 infection in a naive population, not biased by the protective or facilitating immune response established against previous infections.

## MATERIALS AND METHODS

### Ethics statement.

The study protocol was approved by the Ethics Committee for Health of the regional healthcare services of “Serviço de Saúde da Região Autónoma da Madeira (RAM), Entidade Pública Empresarial da RAM” (SESARAM EPERAM) (approval no. 49/2021). All participants gave their informed consent before starting the online questionnaire. All procedures complied with the Declaration of Helsinki of the World Medical Association and regulations for the protection of human subjects, rights, and personal data of the enrolled participants.

### Selection of participants and questionnaire design.

In 2021, we conducted a retrospective questionnaire-based study applied to patients with clinical and/or laboratory diagnoses of dengue fever in 2012–2013 by the regional healthcare services of SESARAM EPERAM, according to WHO diagnostic criteria^4^ and following the guidance of the European Center for Disease Control (ECDC) experts assigned to the Madeira outbreak. According to the protocol established at SESARAM EPERAM during the outbreak, the laboratory diagnosis of dengue was done using different commercial rapid tests for DENV NS1 antigen detection (further details are available in the ECDC 2014 report).^21^ Additionally, reverse transcription polymerase chain reaction was performed for specimens from patients within 7 days from the onset of fever, and IgM and IgG capture ELISA was used for testing patients with fever onset longer than 7 days.^21^ The invited participants were aged between 16 and 62 years at the time of the outbreak.

**Figure 1.**
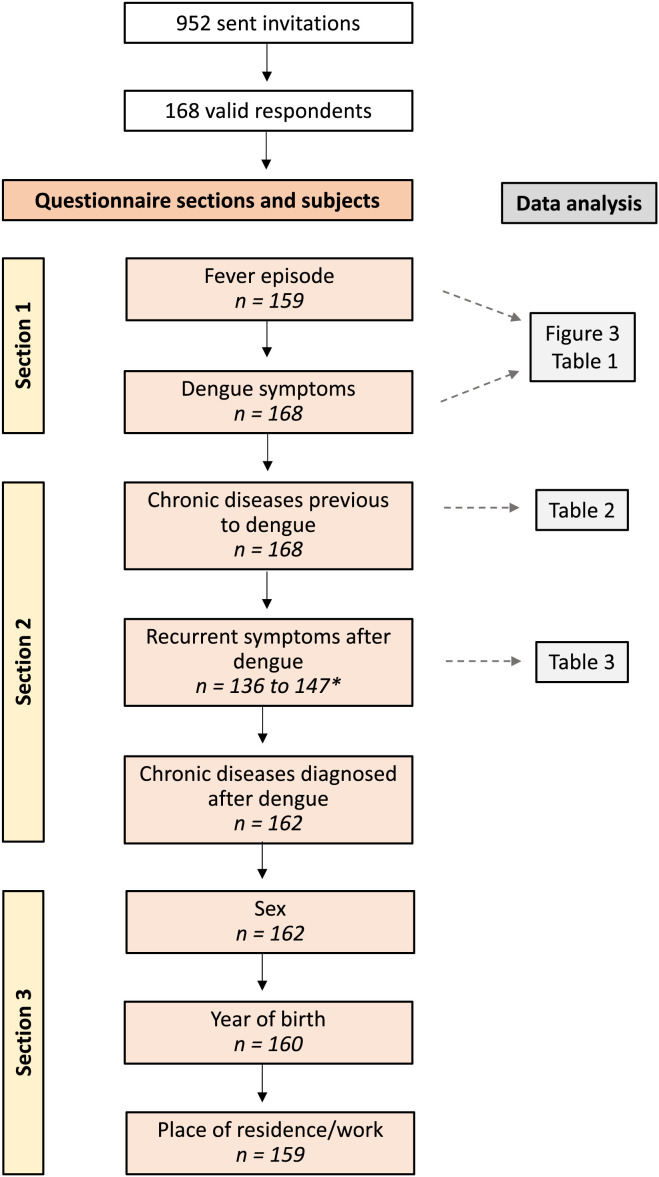
Flow diagram of the sections of the questionnaire and the number of valid participants per subject. * The number of valid participants who responded to each symptom in this question is variable because of the number of responders selecting “I don’t know/I don’t remember” for each symptom.

The questionnaire was newly designed by the authors as a standardized form to collect directly observable parameters, that is, the clinical signs and symptoms experienced during DENV infection and subsequent persistent symptoms or possible sequelae. The multiple-choice options regarding symptoms during the infection and persistent symptoms were based on 1) the more commonly reported clinical parameters in the literature; 2) the findings of García et al.,^14^ to our knowledge the only study reporting long-term persistent symptoms, 2 years postinfection; and 3) the clinical information obtained through a face-to-face questionnaire previously carried out as part of recruitment for a serological study targeting the same population as in the current study. In all questions, an open-field option allowed participants to report other information not included in the multiple options. The questionnaire’s structure, content, and applicability to the studied population were evaluated by a panel of epidemiologists from the Faculty of Medicine of the University of Lisbon and by the SESARAM Internist member of this research work, who was involved in the management of the dengue outbreak of 2012–2013. Questionnaire reliability was determined using Cronbach’s alpha test, and the estimated value for overall items (*alpha* = 0.843) indicated its internal consistency. For the purposes of this study, the questionnaire was structured into three main sections (Figure 1): Section 1 was intended to characterize the signs/symptoms during the infection; Section 2 aimed at evaluating the general health status, before and after the infection, to infer about persistent manifestations after dengue; and Section 3 assessed demographic data of the participants.

## STATISTICAL ANALYSES

The questionnaire was uploaded to the online SurveyMonkey^®^ platform (Momentive Inc., San Mateo, CA), an open-source and cloud-based platform that allows mobile data collection and complies with strict privacy and safety policies. An invitation with a link specifically generated for this questionnaire was sent to the mobile contacts of eligible participants, allowing them to respond anonymously and only once. Completed surveys were reviewed for data entry errors by the senior project members.

Data were extracted in *.xls* format and further edited for analyses in SPSS, version 28.0.0.0 (SPSS Inc., Chicago, IL). For each variable, valid participants were considered as those selecting “yes” or “no,” and participants indicating “I don’t know/won’t answer” or leaving blank fields were excluded. The independence of categorical variables, overall and stratified by sex, was tested by univariate analyses using Pearson’s χ^2^ tests (or Fisher’s exact tests, when at least one of the expected values was <5). Odds ratio (OR) and respective 95% CI were calculated only for significant associations. Mean age differences, overall and per sex, were assessed by Mann–Whitney (or *t*-tests, according to the results of Shapiro–Wilk tests). Logistic regression was used to identify predictive variables for symptom persistence. All results were interpreted at a significance level of 0.05.

## RESULTS

### Characterization of participants.

**Figure 2.**
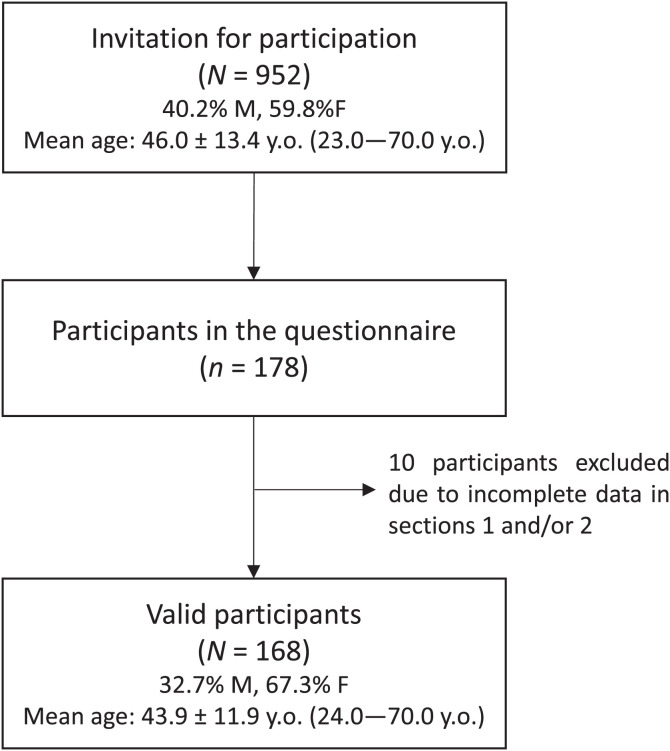
Flow diagram of participants’ recruitment.

The invitation for participation in the survey was sent to 952 contacts (40.2% male, 59.8% female; aged 23–70 years, mean age 46.0 ± 13.4 years (equivalent to a mean age of 38.0 years at the time of the outbreak), ascertained from the SESARAM EPERAM database as having had clinical and/or laboratory diagnosis of dengue fever during the outbreak. Of the 178 participants, not all were considered valid: a few participants did not answer the survey sections referring to signs/symptoms at the time of the infection (*n =* 6), to the general health status before the infection (*n =* 6), and/or to recurrent signs and symptoms after the infection (*n =* 10), which were considered relevant for the purpose of our study and were therefore excluded from the analyses. Thus, 168 individuals, most of Portuguese nationality (98.1%), were considered valid participants. In terms of sex (32.7% male, 67.3% female) and age range (aged 24–70 years; mean age 43.9 ± 11.9 years, equivalent to a mean age of 35.9 years at the time of the outbreak) (Figure 2), this is a representative subset of the invited participants because no significant differences were found when testing for proportions and means (both *P*-values >0.05).

### Dengue clinical manifestations.

**Figure 3.**
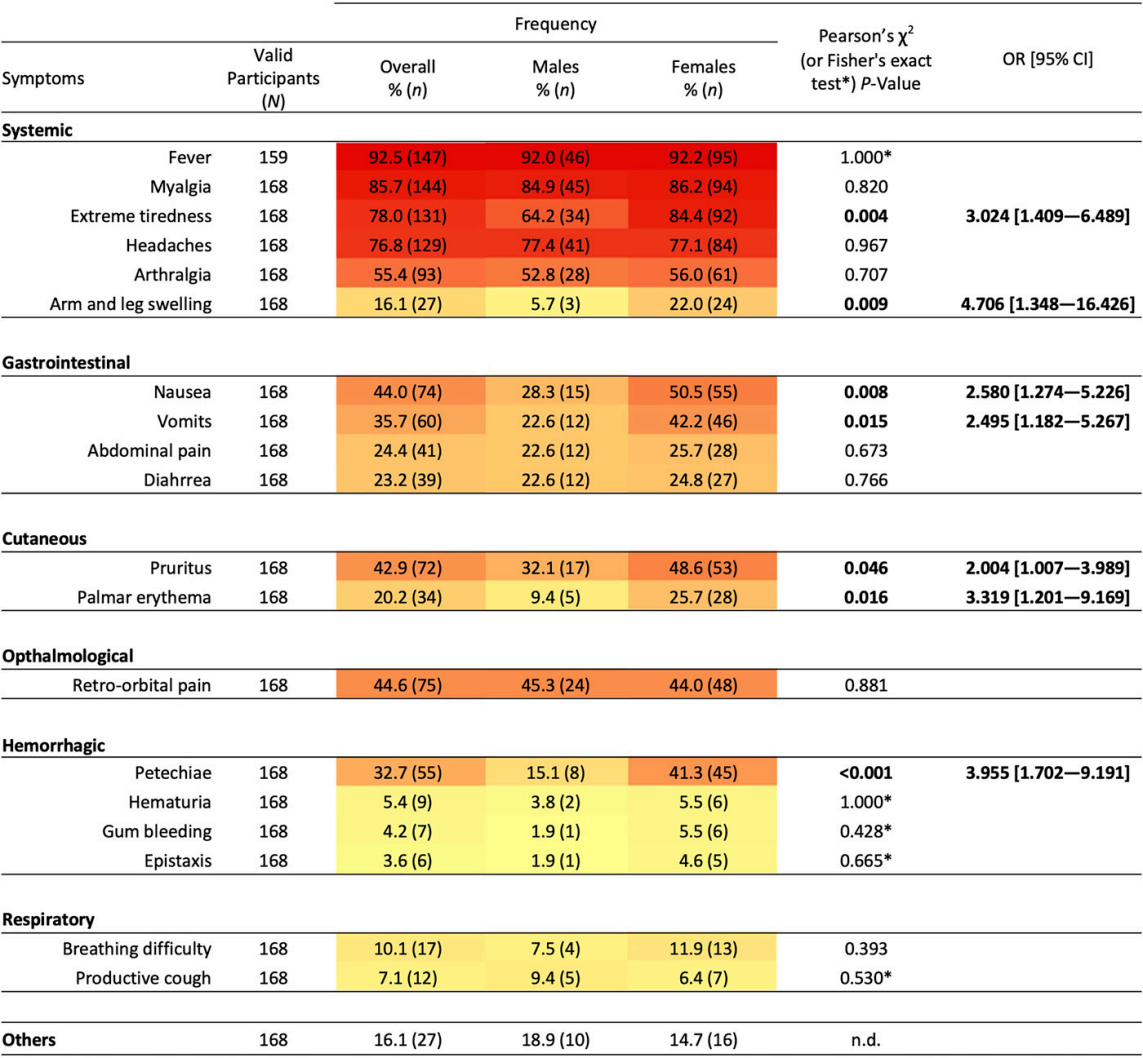
Frequency of participants reporting different symptoms at the time of dengue (overall and per sex). The “Others” category includes loss of appetite, weight loss, ageusia, anosmia, loss of visual acuity, chest pain, and loss of consciousness. Pearson χ^2^ or Fisher’s exact tests were used, with Fisher’s *P*-values (*) shown when at least one of the expected values was <5; odds ratios (ORs) and 95% CIs are indicated only for variables found to be significant (in bold); n.d. = not determined.

During the active infection, 92.5% of the participants reported a fever episode for which 98.0% sought medical assistance. Among other symptoms, the most frequently reported were systemic symptoms such as myalgia (85.7%), extreme tiredness (78.0%), headaches (76.8%), and arthralgia (55.4%), whereas hemorrhagic manifestations such as hematuria (5.4%), gum bleeding (4.2%), and epistaxis (3.6%) were less frequently stated (Figure 3). An unexpectedly high frequency of petechiae (32.7%) was also reported. Significantly different frequencies of signs/symptoms were observed according to sex, with pruritus, vomiting, nausea, extreme tiredness, palmar erythema, petechiae, and arm and leg swelling being 2.0- to 4.7-fold more frequent in females than in males (*P* ≤0.046; Figure 3).

When comparing the age distribution of participants exhibiting or not exhibiting each symptom, significant differences were found only for arm and leg swelling, which was more common in older participants (40.1 ± 12.0 years versus 35.1 ± 11.8 years, *P* = 0.037; Table 1). No significant differences were observed for the mean age of males and females presenting with each symptom (Table 1).

**Table 1:** Mean age of participants reporting or not reporting each symptom at the time of dengue infection (overall and per sex)

Symptoms	Valid Participants (*N*)	Participants with Symptom			*t*-Test (or Mann–Whitney*) *P*-Value	Participants Without Symptom	
		Overall, Years ± SD (*n*)	Males, Years ± SD (*n*)	Females, Years ± SD (*n*)		Overall, Years ± SD	*t-*Test (or Mann–Whitney*) *P*-Value^†^
Systemic							
Fever	152	35.8 ± 12.2 (141)	37.2 ± 12.2 (46)	35.1 ± 12.1 (95)	0.330	40.1 ± 10.7 (11)	0.258
Myalgia	160	35.2 ± 11.9 (137)	36.3 ± 11.9 (45)	34.7 ± 12.0 (93)	0.448	40.0 ± 11.2 (23)	0.072
Extreme Tiredness	160	35.1 ± 12.5 (124)	36.9 ± 12.8 (34)	34.4 ± 12.4 (91)	0.330	38.8 ± 9.3 (36)	0.094
Headaches	160	35.7 ± 11.9 (124)	37.6 ± 12.4 (41)	34.7 ± 11.6 (83)	0.201	36.8 ± 12.1 (36)	0.631
Arthralgia	160	36.8 ± 11.7 (89)	36.3 ± 10.9 (28)	37.1 ± 12.1 (61)	0.782	34.7 ± 12.2 (71)	0.273
Arm and Leg Swelling	160	40.1 ± 12.0 (27)	39.3 ± 5.9 (3)	40.2 ± 12.6 (24)	0.856*	35.1 ± 11.8 (133)	0.037*
Gastrointestinal							
Nausea	160	36.1 ± 12.5 (69)	37.1 ± 11.6 (15)	35.9 ± 12.9 (54)	0.746	36.1 ± 12.5 (69)	0.837
Vomits	160	34.0 ± 12.9 (57)	36.8 ± 12.4 (12)	33.3 ± 13.0 (45)	0.409	37.0 ± 11.3 (103)	0.165*
Abdominal Pain	160	35.3 ± 13.4 (39)	34.8 ± 12.0 (12)	35.5 ± 14.2 (27)	0.885	36.1 ± 11.4 (121)	0.719
Diarrhea	160	34.1 ± 12.8 (38)	37.4 ± 12.3 (12)	32.6 ± 13.0 (26)	0.285	36.5 ± 11.6 (122)	0.241*
Cutaneous							
Pruritus	160	37.4 ± 12.3 (68)	37.1 ± 13.5 (17)	37.5 ± 12.1 (52)	0.902	34.8 ± 11.6 (92)	0.174
Palmar Erythema	160	36.9 ± 12.0 (32)	40.8 ± 5.8 (5)	36.1 ± 12.7 (27)	0.434	35.7 ± 11.9 (128)	0.609
Ophthalmological							
Retro-orbital Pain	160	36.5 ± 11.5 (71)	38.5 ± 9.7 (24)	35.5 ± 12.2 (47)	0.298	35.4 ± 12.3 (89)	0.570
Hemorrhagic							
Petechiae	160	33.7 ± 12.1 (52)	32.8 ± 11.7 (8)	33.9 ± 12.3 (44)	0.806	37.0 ± 11.7 (108)	0.121*
Hematuria	160	37.7 ± 16.0 (7)	49.0 ± 1.4 (2)	33.2 ± 17.1 (5)	0.381*	35.8 ± 11.8 (153)	0.683
Gum Bleeding	160	43.0 ± 11.9 (6)	48.0 (1)	42.0 ± 13.0 (5)	1.000*	35.6 ± 11.9 (154)	0.138
Epistaxis	160	36.2 ± 16.9 (5)	48.0 (1)	33.3 ± 18.0 (4)	0.800*	35.9 ± 11.8 (155)	0.956
Respiratory							
Breathing Difficulty	160	38.2 ± 13.8 (17)	37.8 ± 14.2 (4)	38.4 ± 14.3 (13)	1.000*	35.6 ± 11.7 (143)	0.396
Productive Cough	160	35.7 ± 13.1 (12)	37.8 ± 17.2 (5)	34.1 ± 10.5 (7)	0.656*	35.9 ± 11.9 (148)	0.943
Others	160	39.2 ± 13.4 (26)	38.9 ± 12.2 (10)	39.4 ± 14.4 (16)	n.d.	35.3 ± 11.6 (134)	n.d.

n.d. = not determined.

The “Others” category includes loss of appetite, weight loss, ageusia, anosmia, loss of visual acuity, chest pain, and loss of consciousness. *t*-test or Mann–Whitney (*) tests were used according to the normality of distribution accessed by Shapiro–Wilk tests. Significant *P*-values are highlighted in bold.

^†^ To compare mean age of participants with and without symptoms.

### Health status before dengue.

Participants were asked about the existence of chronic diseases before dengue in 2012–2013. Most (72.6%, *n =* 122) affirmed not having a diagnosis of chronic diseases at that time, whereas 27.4% (*n =* 46) had one or more chronic diseases (Table 2). The most frequently reported comorbidities by our participants were hypertension (*n =* 16), diabetes (*n =* 7), and respiratory disease (*n =* 5). It was also noted that, as expected, the mean age of participants with chronic diseases (41.2 ± 11.4 years) was significantly higher than those without chronic diseases (34.1 ± 11.6 years; *P* <0.001). Also noteworthy, the mean age of participants with respiratory and autoimmune diseases was much lower (<30 years) than the mean age for other chronic diseases (over 44 years). Hypertension was the most reported chronic disease, and therefore we tested the association of this condition with each symptom during dengue by comparing their frequencies in hypertensive and nonhypertensive participants. For these comparisons, hypertension was suggested as a risk factor for arm and leg swelling (*P* = 0.025, OR = 3.743, 95% CI: 1.231–11.380) but protective against retro-orbital pain (*P* = 0.029, OR = 0.256, 95% CI: 0.070–0.936; data not shown).

**Table 2:** Frequency and mean age of participants reporting chronic disease before dengue in 2012–2013

Chronic Disease before Dengue	Frequency % (*n*)	Mean Age, Years ± SD (*n*)
Without Previous Chronic Disease	72.6 (122)	34.1 ± 11.6 (119)
With Previous Chronic Disease	27.4 (46)	41.2 ± 11.3 (45)
Hypertension	9.5 (16)	44.1 ± 11.3 (16)
Diabetes	4.2 (7)	52.1 ± 8.6 (7)
Respiratory Disease	3.0 (5)	29.0 ± 11.1 (5)
Hypercholesterolemia	1.8 (3)	48.3 ± 9.5 (3)
Autoimmune Disease	1.8 (3)	24.5 ± 12.0 (2)
Heart Disease	1.2 (2)	49.5 ± 10.6 (2)
Others	8.9 (15)	–

The “Others” category includes thrombin III deficiency, ulcerative colitis, kidney disease, venous thromboembolism, ophthalmological disease, and other nonspecified chronic diseases.

### Long-term persistence of clinical symptoms after dengue.

In Section 2 of the questionnaire, participants were asked about persistent symptoms after dengue, among those symptoms shown in Table 3. To distinguish between occasional symptoms of unspecific cause and recurrent clinical manifestations that could point to sequelae or chronic health problems, we centered our attention on previously nonexistent or rare symptoms that became frequently reported (several times per year) after dengue. Considering that previous chronic diseases may influence the type and frequency of post-dengue recurrent symptoms, participants reporting comorbidities with onset before 2012–2013 were excluded from this analysis. Note the lower response rate in this and the following sections, with dropout of nine participants (four with previous chronic disease and five without previous chronic disease; Figure 1).

**Table 3:** Frequency and mean age of participants reporting recurrent symptoms after dengue (overall and per sex)

Recurrent Symptoms after Dengue	Frequency						Mean Age at the Time of the Survey		
	Valid Part.	Overall, % (*n*)	M, % (*n*)	F, % (*n*)	Pearson’s χ^2^ (or Fisher’s exact test*) *P*-Value	OR (95% CI)	Part. with Symptoms, Years ± SD (*n*)	Part. without Symptoms, Years ± SD (*n*)	*t*-Test (or Mann–Whitney*) *P*-Value^†^
Headaches	107	35.5 (38)	20.6 (7)	42.5 (31)	**0.028**	**2.847 (1.099–7.377)**	37.1 ± 10.9 (38)	44.0 ± 12.2 (69)	0.029*
Abundant Hair Loss	107	30.8 (33)	15.1 (5)	37.8 (28)	**0.019**	**3.409 (1.180–9.851)**	39.5 ± 12.8 (33)	43.0 ± 11.9 (74)	0.311*
Extreme Tiredness	109	29.4 (32)	17.6 (6)	34.7 (26)	0.071	–	46.2 ± 13.8 (32)	40.6 ± 11.4 (77)	0.092
Arthralgia	105	25.7 (27)	23.5 (8)	26.8 (19)	0.723	–	50.5 ± 14.7 (27)	39.6 ± 10.4 (78)	**<0.001**
Myalgia	103	25.2 (26)	13.3 (4)	30.1 (22)	0.074	–	45.2 ± 15.3 (26)	41.0 ± 11.0 (77)	0.322
Bone Pain	105	20.0 (21)	11.8 (4)	23.9 (17)	0.144	–	54.5 ± 11.2 (21)	39.0 ± 10.5 (84)	<0.001*
Palpitations	102	16.7 (17)	12.5 (4)	18.6 (13)	0.445	–	39.3 ± 13.1 (17)	42.5 ± 12.1 (85)	0.455
Blurred Vision	106	16.0 (17)	14.3 (5)	16.9 (12)	0.730	–	43.3 ± 13.3 (17)	41.8 ± 12.1 (89)	0.953
Memory Loss	99	14.1 (14)	12.5 (4)	14.9 (10)	1.000*	–	44.0 ± 11.8 (14)	41.7 ± 12.3 (85)	0.600*
Loss of Appetite	105	10.5 (11)	2.9 (1)	14.1 (10)	0.099*	–	49.8 ± 14.5 (11)	41.1 ± 11.7 (94)	0.197*
Retroorbital Pain	104	9.6 (10)	8.6 (3)	10.1 (7)	1.000*	–	39.2 ± 15.0 (10)	42.2 ± 12.1 (94)	0.563*

F = female; M = male; OR = odds ratio; Part. = participants.

Participants with previous chronic disease (*n =* 46) were not considered in this analysis. Pearson χ^2^ or Fisher’s exact tests were used for the frequency comparison among sexes, with Fisher’s *P* values (*) shown when at least one of the expected values was <5. ORs and 95% CI are indicated only for variables found to be significant. Significant *P*-values are highlighted in bold.

^†^ To compare the mean age of participants with and without symptoms, *t*-test or Mann–Whitney test were used, according to the normality of distribution accessed by Shapiro–Wilk tests.

Of the 117 valid participants, 61.5% (*n =* 72) reported at least one long-term persistent symptom after dengue, 68.1% (*n =* 49) of which sought medical assistance due to these symptoms. Systemic manifestations such as headaches (35.5%), abundant hair loss (30.8%), extreme tiredness (29.4%), arthralgia (25.7%), and myalgia (25.2%) were reported by more than one-quarter of these participants (Table 3). Also noteworthy, among participants with persistent symptoms, 18.1% (*n =* 13) described the onset of chronic illness within the 4-year period after dengue, with the most frequent being ophthalmological (*n =* 4) and autoimmune (*n =* 4) disorders (data not shown). On the other hand, among participants without persistent symptoms, only 2.2% (*n =* 1) developed a chronic autoimmune disorder, which was significantly different (*P* = 0.008, OR = 9.565, 95% CI: 1.584–212.400) compared with the onset of chronic diseases in participants with persistent symptoms.

Additionally, female sex was suggested as a risk factor for the persistence of at least one symptom in our dataset because these were reported by 72.6% of females compared with 51.0% of males (*P* = 0.012, OR = 2.553, 95% CI: 1.273–5.118). On the other hand, the mean age of participants did not seem to influence the long-term persistence of at least one symptom. When assessing the frequencies of each recurrent symptom according to sex, we found significant differences in headaches (*P* = 0.028, OR = 2.847, 95% CI: 1.099–7.377) and abundant hair loss (*P* = 0.019, OR = 3.409, 95% CI: 1.180-9.851), with a higher frequency in females (Table 3). Additionally, the mean age of participants was found to correlate with persistent headaches (*P* = 0.029), which were more common in younger patients, whereas arthralgia (*P* <0.001) and bone pain (*P* <0.001) prevailed in older patients (Table 3).

When testing the association of symptoms at the time of active infection as prognostic factors for the long-term persistence of at least one symptom, we found by univariate analyses that breathing difficulties were associated with a higher risk of persistence: 93.8% of participants with breathing difficulties versus 62.4% of participants without breathing difficulties confirmed having at least one persistent symptom (*P* = 0.012, OR = 9.034, 95% CI: 1.160–70.368, data not shown). The logistic regression model suggests that breathing difficulties during the infection and female sex are predictors of symptoms’ persistence (*P* = 0.033, OR = 9.489, 95% CI: 1.195–75.297 and *P* = 0.009, OR = 2.670, 95% CI: 1.275–5.589, respectively), corroborating the results of the univariate analyses.

## DISCUSSION

Here we report the results of an online questionnaire applied to symptomatic dengue patients in Madeira Island, with clinical diagnosis in 2012–2013, at the time of the single dengue outbreak in this population. According to the records of healthcare services, seeking medical assistance primarily occurred during the febrile period of dengue infection, leading to an underestimated clinical picture of the disease because some dengue manifestations only appear later. Additionally, because most of the patients were not followed up, no registry of expanded manifestations exists beyond the acute phase of the infection. Our questionnaire aimed to characterize symptoms retrospectively during active infection and to assess health status before and after dengue diagnosis, identifying long-term manifestations that may be triggered by dengue. As mentioned earlier, we expected self-reported manifestations to be a good representation of those verified at first exposure to the DENV1 serotype that were not biased by immune responses mounted against previous DENV infections. Our results suggest that symptom persistence after primary dengue might be more frequent and longer lasting than anticipated, with a potential impact on the health status and well-being of the affected patients.

The most frequent symptoms during the clinical period were fever, myalgia, extreme tiredness, and headaches, reported by more than three-quarters of our participants, whereas vomiting, pruritus, nausea, retro-orbital pain, and arthralgia occurred in 50% to 35% of participants. In a subset (*n =* 67) of this same infected population, Freitas et al.^23^ described similar clinical manifestations as the most common. However, their frequencies cannot be directly compared with our study because they referred only to hospitalized patients and to the most relevant symptoms motivating their search for medical care and/or whose onset occurred during hospitalization. Conversely, our study included nonhospitalized participants and retrospectively analyzed the entire period of symptoms, from the early-acute to the convalescence phase. It is important to stress that symptoms are known to vary during the infection, and some even worsen after the acute phase.^6^^,^^10^^–^^12^^,^^24^ Regarding the systemic and musculoskeletal symptoms, our results are in line with previous studies in other populations.^6^^–^^8^^,^^10^^–^^13^^,^^24^^–^^29^ It is noteworthy, however, that gastrointestinal symptoms were 1.5- to 2.7-fold less frequent in our study compared with others.^7^^,^^8^^,^^10^^,^^12^^,^^13^^,^^24^^,^^25^^,^^28^ Hemorrhagic manifestations, when considered altogether, were mentioned by our participants at similar or slightly lower proportions than in other studies.^6^^,^^8^^,^^29^ However, marked differences between studies are found when hemorrhagic signs are analyzed individually. For example, gum bleeding and epistaxis were 1.6- to 4.2-fold less frequent in our study, whereas petechiae and hematuria were 1.4- to 5-fold more frequent in our study than in previous research.^6^^,^^7^^,^^28^^,^^29^ In 2015 and 2020, primary DENV1 local transmission in nonendemic European regions occurred in France^30^ and Italy,^31^ respectively, and both described similar clinical outcomes to our study. Nevertheless, direct comparisons to our study are impossible because of their small number of cases (*N* ≤12). In Effler et al.,^29^ which included a naive population in Hawaii facing a DENV1 outbreak, most symptoms were 1.4- to 2.2-fold more frequent than in our study, including hemorrhagic symptoms (except for petechiae, which were found at slightly lower proportions). We recognize that there might be an overestimation of petechiae in our study (32.7%) because, from our previous experience with a face-to-face questionnaire, participants tend to misinterpret these as other nonhemorrhagic cutaneous manifestations, and thus their frequency should be interpreted with caution.

Gastrointestinal and hemorrhagic manifestations are considered warning signs and predictors of severity, more commonly present in secondary infections, and known to be influenced by DENV serotype/genotype (reviewed in Yuan et al.).^32^ Nevertheless, mild hemorrhagic manifestations have been observed in uncomplicated dengue, including in primary infections.^29^^,^^33^ The results of different studies, including those presented here, suggest therefore that hemorrhagic manifestations in primary infections may be more common than anticipated. As for differences between studies addressing symptoms during the infection, we must consider that most studies were held in endemic areas and do not discriminate between primary and secondary dengue, whereas our data are compatible with an expected milder clinical outcome in primary infections. Furthermore, their different designs, including age and sex distribution, forms of assessment and duration of follow-up, and differences in DENV serotypes and host population backgrounds, with distinct genetics and immunological experiences (introduced by natural exposure and vaccination plans), hinder direct comparison.

Most of our participants (72.6%) reported not having any chronic diseases before dengue, what is concurrent with the findings of Martelli et al.,^8^ where 85.4% of the participants perceived their general health status before the infection as being very good or good, but discordant with Teixeira et al.,^13^ in which 55.8% of the participants had previous comorbidities. The most frequently reported chronic diseases in our cohort are also not surprising because these are among the most prevalent in our population background.^34^ When investigating possible correlations between previous chronic disease and each dengue symptom, hypertension was found to correlate significantly with an increased risk for arm and leg swelling but was protective against retro-orbital pain. Apart from the influence of hypertension in the progression of the viral infection to severe forms of dengue,^35^ likely potentiated by the pro-inflammatory state of hypertense patients,^36^ peripheral edema can also be explained by the condition itself,^37^ and/or side effects of antihypertensive drugs.^38^ We here also hypothesize about the influence of antihypertensive drugs in reducing retro-orbital pain in dengue patients under these therapeutics because several of these drugs have been shown to reduce headaches, given their effects on vascular tone in the cerebrovascular circulation.^39^

Persistence of clinical manifestations after dengue has been previously reported and may be caused by systemic damage during the acute phase and/or host-related factors affecting their recovery. However, their prevalence and duration vary widely in the literature, ranging from 9% to 57% of the participants reporting at least one recurrent symptom, lasting from 1 month up to 2 years.^6^^,^^10^^–^^14^^,^^24^ In our study, 61.5% of the participants reported at least one persistent symptom in the 8 years after dengue, of which 68.1% sought medical assistance, thus representing a measure of intensity/persistence of their symptoms. Most of the existing literature describes similar persistent symptoms to those reported in our study, although generally at much lower frequencies. For instance, persistent headaches were reported by 35.5% of our participants, but only 2.1% up to 18.9% of the participants of several other studies, at different time points within 1 to 6 months after dengue.^6^^,^^10^^–^^13^^,^^24^ In some of these studies, abundant hair loss ranged from 2.1% to 19.5%,^6^^,^^10^^,^^13^^,^^24^ whereas it reached 30.8% in this study. Also, in previous studies, persistent arthralgia and retroorbital pain represented, respectively, 4.5–11.5%,^6^^,^^10^^,^^13^^,^^24^ and 1.1–3.0%,^10^^,^^11^^,^^24^ whereas these were described by 25.7% and 9.6% of our participants. Previous studies also indicate a resolution of symptoms in most patients within a few months after infection (persistence rates of 8.5–33.3% at 1–2 months^10^^–^^12^^,^^24^ and 7.6–11.5% at 6 months^6^^,^^13^), ending their follow-up after these time windows. As mentioned before, discrepancies between studies can be due to myriad factors, such as differences in study design (age and sex distribution, forms of assessment, and time of follow-up), but also due to biological effects, such as differences in virulence, viral load, and tissue tropism between DENV serotypes, and host comorbidities, immunological status, and genetic susceptibility. Furthermore, the perception of health status may be variable in different populational and cultural backgrounds.

To our knowledge, the only study reporting long-term persistent symptoms is that of García et al.,^14^ in which 56.7% of Cuban participants had at least one chronic symptom 2 years after dengue. Their findings are similar to ours, except for differences noted for persistent headaches, abundant hair loss (respectively, 2.4- and 3.3-fold less frequent than in our study), and retroorbital pain (1.5-fold more frequent than in our study). We should stress that the García et al. study^14^ was undertaken in a dengue-endemic area, whereas ours refers to primary dengue. In this context, our results suggest that, even in naive populations, the occurrence of persistent symptoms may be more frequent than previously thought and such symptoms may prevail for several years. Nearly 20% of our participants have described persistent ophthalmological signs such as blurred vision and/or retroorbital pains in the years after dengue, four of which have developed ophthalmologic chronic disease. There is, in fact, an increasing body of evidence about dengue-associated eye disease, the onset of which has been described to occur immediately or a few months after the infection (reviewed in Ng and Teoh 2015).^40^

Also noteworthy, our data are, to our knowledge, the first to investigate and suggest sex and age as good predictors of specific symptoms, either during the infection period or persistent symptoms. During the infection, pruritus, vomiting, nausea, extreme tiredness, palmar erythema, petechiae, and arm and leg swelling were significantly more frequent in females, and arm and leg swelling were more common in older participants. The effect of these variables in an active infection is likely due to hormonal and psychosomatic differences and/or the co-occurrence of age-related conditions. As for persistent symptoms, headaches, and abundant hair loss were more common in females, whereas headaches were more frequent in younger participants, and arthralgia and bone pain in older participants. Although there is no consensus about the physiopathological mechanisms underlying the persistence of symptoms, it has been suggested that the excessive cytokine production during the acute phase^41^ may cause neuroendocrine, musculoskeletal, and immunological damage,^42^ which may affect males and females in different ways and lead to more durable consequences in older patients, given their lower regenerative capacity of affected tissues.

Nevertheless, the results presented here should be approached with caution due to a few limitations of our study. All participants had clinical diagnosis of dengue according to the WHO,^4^ following the recommendations of ECDC to the regional healthcare authorities, but given the anonymous nature of the questionnaire, it is not possible to estimate the proportion of participants with laboratory-confirmed dengue. Therefore, we must consider that our results may be biased by misdiagnosed cases, caused by other infectious or noninfectious health conditions. Nevertheless, an ongoing study by our team in a subset (*n =* 119) of invited participants showed that 8 years after the outbreak, more than 90% were positive for ELISA anti-DENV IgG and foci reduction neutralization test (Henriques et al., submitted for publication). This is in accordance with publications in nonendemic areas, reporting a loss of seropositivity in adults, of approximately 4% per 3-year period^43^^,^^44^; and indicates that most of our participants were, indeed, true dengue cases. Also, we must consider the subjectivity of self-reported data, possible misinterpretation of the questions/multiple answers, and a higher representation of participants with more severe complaints. Moreover, data reports to a reference period from 2012 to 2021, which introduces recall bias. Lastly, we recognize that a sex- and age-matched, noninfected, asymptomatic, or population-based control sample would have been relevant for distinguishing persistent symptoms resulting from dengue from those naturally occurring in the population. However, because our study was undertaken several years after the outbreak, we found it unlikely, and prone to recall bias, that a sample set not affected by symptomatic dengue could identify recurrent previously nonexistent symptoms.

In sum, despite its limitations, this study’s findings contribute to the existing body of knowledge with a description of the symptoms during primary dengue in a naive population where a single DENV1 outbreak occurred. Our study also illustrates that even in a primary infection, dengue may have significant implications on the health status and well-being of a considerable proportion of the infected population because recurrent symptoms may persist for several years. These data can therefore be considered in terms of healthcare policies, guiding new procedures and strategies for disease control and prevention, and strengthening the importance of the clinical follow-up of dengue patients.

## References

[1] Wilder-Smith A Ooi E-E Horstick O Wills B, 2019. Dengue. Lancet 393: 350–363. 30696575 10.1016/S0140-6736(18)32560-1

[2] Bhatt S , 2013. The global distribution and burden of dengue. Nature 496: 504–507. 23563266 10.1038/nature12060 PMC3651993

[3] Balmaseda A , 2010. Trends in patterns of dengue transmission over 4 years in a pediatric cohort study in Nicaragua. J Infect Dis 201: 5–14. 19929380 10.1086/648592 PMC3724236

[4] World Health Organization , 2009. Dengue Guidelines for Diagnosis, Treatment, Prevention, and Control: New Edition (No. WHO/HTM/NTD/DEN/2009.1). Geneva, Switzerland: WHO. 23762963

[5] Elson WH , 2020. Measuring health-related quality of life for dengue patients in Iquitos, Peru. PLoS Negl Trop Dis 14: e0008477. 32722709 10.1371/journal.pntd.0008477 PMC7413550

[6] Tiga-Loza DC Martínez-Vega RA Undurraga EA Tschampl CA Shepard DS Ramos-Castañeda J, 2020. Persistence of symptoms in dengue patients: A clinical cohort study. Trans R Soc Trop Med Hyg 114: 355–364. 32125417 10.1093/trstmh/traa007

[7] da Silva NS Undurraga EA da Silva Ferreira ER Estofolete CF Nogueira ML, 2018. Clinical, laboratory, and demographic determinants of hospitalization due to dengue in 7613 patients: A retrospective study based on hierarchical models. Acta Trop 177: 25–31. 28964768 10.1016/j.actatropica.2017.09.025

[8] Martelli CMT Nascimento NE Suaya JA Siqueira JB Souza WV Turchi MD Guilarde AO Peres JB Shepard DS, 2011. Quality of life among adults with confirmed dengue in Brazil. Am J Trop Med Hyg 85: 732–738. 21976580 10.4269/ajtmh.2011.11-0067 PMC3183785

[9] World Health Organization , 1997. Dengue Haemorrhagic Fever: Diagnosis, Treatment, Prevention, and Control. 2nd edition. Geneva, Switzerland: WHO.

[10] Dettogni RS Tristão-Sá R dos Santos M da Silva FF Louro ID, 2015. Single nucleotide polymorphisms in immune system genes and their association with clinical symptoms persistence in dengue-infected persons. Hum Immunol 76: 717–723. 26429310 10.1016/j.humimm.2015.09.026

[11] Teixeira L de AS Lopes JSM Martins AGdC Campos FAB Miranzi SdSC Nascentes GAN, 2010. Persistência dos sintomas de dengue em uma população de Uberaba, Minas Gerais, Brasil. Cad Saude Publica 26: 624–630. 20464080 10.1590/s0102-311x2010000300019

[12] Seet RCS Quek AML Lim ECH, 2007. Post-infectious fatigue syndrome in dengue infection. J Clin Virol 38: 1–6. 17137834 10.1016/j.jcv.2006.10.011

[13] Teixeira LdAS Nogueira FPdS Nascentes GAN, 2017. Prospective study of patients with persistent symptoms of dengue in Brazil. Rev Inst Med Trop São Paulo 59: e65. 28876417 10.1590/S1678-9946201759065 PMC5587034

[14] García G , 2011. Long-term persistence of clinical symptoms in dengue-infected persons and its association with immunological disorders. Int J Infect Dis 15: e38–e43. 21112804 10.1016/j.ijid.2010.09.008

[15] Fabre A Couvelard A Degott C Lagorce-Pagès C Bruneel F Bouvet E Vachon F, 2001. Dengue virus induced hepatitis with chronic calcific changes. Gut 49: 864–865. 11709524 10.1136/gut.49.6.864 PMC1728549

[16] Rahim A Hameed A Ishaq U Malik J Zaidi SMJ Khurshid H Malik A Satti DI Naz H, 2022. Cardiovascular sequelae of dengue fever: A systematic review. Expert Rev Cardiovasc Ther 20: 465–479. 35612830 10.1080/14779072.2022.2082945

[17] Trivedi S Chakravarty A, 2022. Neurological complications of dengue fever. Curr Neurol Neurosci Rep 22: 515–529. 35727463 10.1007/s11910-022-01213-7 PMC9210046

[18] Sigera PC Rajapakse S Weeratunga P De Silva NL Gomes L Malavige GN Rodrigo C Fernando SD, 2020. Dengue and post-infection fatigue: Findings from a prospective cohort – The Colombo Dengue Study. Trans R Soc Trop Med Hyg 115: 669–676. 10.1093/trstmh/traa110 PMC9115379 33099653

[19] Garcia MN Hause AM Walker CM Orange JS Hasbun R Murray KO, 2014. Evaluation of prolonged fatigue post–West Nile virus infection and association of fatigue with elevated antiviral and proinflammatory cytokines. Viral Immunol 27: 327–333. 25062274 10.1089/vim.2014.0035 PMC4150370

[20] Parra B , 2016. Guillain–Barré syndrome associated with zika virus infection in Colombia. N Engl J Med 375: 1513–1523. 27705091 10.1056/NEJMoa1605564

[21] European Centre for Disease Prevention and Control, Secretaria Regional dos Assuntos Sociais , 2014. Dengue Outbreak in Madeira, Portugal, March 2013. Stockholm, Sweden: ECDC.

[22] Thomas EA John M Kanish B, 2010. Mucocutaneous manifestations of dengue fever. Indian J Dermatol 55: 79–85. 20418984 10.4103/0019-5154.60359 PMC2856380

[23] Freitas TE Henriques T Chaves S Silva S Freitas PR Brazão ML, 2014. Dengue: caracterização clínico-laboratorial dos doentes internados durante a 1^a^ epidemia europeia do séc XXI e revisão da literatura. Med Interna (Bucur) 21: 6–11.

[24] Tristão-Sá R , 2012. Clinical and hepatic evaluation in adult dengue patients: A prospective two-month cohort study. Rev Soc Bras Med Trop 45: 675–681. 23295867 10.1590/s0037-86822012000600004

[25] Hasan MJ Tabassum T Sharif M Khan Md AS Bipasha AR Basher A Islam MR Amin MR, 2021. Comparison of clinical manifestation of dengue fever in Bangladesh: An observation over a decade. BMC Infect Dis 21: 1113. 34715814 10.1186/s12879-021-06788-z PMC8555248

[26] Lim JK , 2020. Clinical and epidemiologic characteristics associated with dengue fever in Mombasa, Kenya. Int J Infect Dis 100: 207–215. 32891734 10.1016/j.ijid.2020.08.074 PMC7670221

[27] Bonifay T Vesin G Bidaud B Bonnefoy C Dueymes M Nacher M Djossou F Epelboin L, 2019. Clinical characteristics and predictive score of dengue vs. chikungunya virus infections. Med Mal Infect 49: 250–256. 30348472 10.1016/j.medmal.2018.09.010

[28] Jain S , 2017. Predictors of dengue-related mortality and disease severity in a tertiary care center in north India. Open Forum Infect Dis 4: ofx056. 28491893 10.1093/ofid/ofx056 PMC5419201

[29] Effler PV , 2005. Dengue fever, Hawaii, 2001–2002. Emerg Infect Dis 11: 742–749. 15890132 10.3201/eid1105.041063 PMC3320380

[30] Succo T , 2016. Autochthonous dengue outbreak in Nîmes, south of France, July to September 2015. Euro Surveill 21: 30240. 10.2807/1560-7917.ES.2016.21.21.30240 27254729

[31] Barzon L , 2021. Autochthonous dengue outbreak in Italy 2020: Clinical, virological, and entomological findings. J Travel Med 28: taab130. 34409443 10.1093/jtm/taab130 PMC8499737

[32] Yuan K Chen Y Zhong M Lin Y Liu L, 2022. Risk and predictive factors for severe dengue infection: A systematic review and meta-analysis. PLoS One 17: e0267186. 35427400 10.1371/journal.pone.0267186 PMC9012395

[33] Anantapreecha S Chanama S A-Nuegoonpipat A Naemkhunthot S Sa-Ngasang A Sawanpanyalert P Kurane I, 2005. Serological and virological features of dengue fever and dengue haemorrhagic fever in Thailand from 1999 to 2002. Epidemiol Infect 133: 503–507. 15962557 10.1017/s0950268804003541 PMC2870274

[34] Romana GQ Kislaya I Salvador MR Gonçalves SC Nunes B Dias C, 2019. Multimorbilidade em Portugal: Dados do Primeiro Inquérito Nacional de Saúde com Exame Físico. Acta Med Port 32: 30–37. 30753801 10.20344/amp.11227

[35] Ng WY , 2022. A double whammy: The association between comorbidities and severe dengue among adult patients – A matched case-control study. PLoS One 17: e0273071. 36126060 10.1371/journal.pone.0273071 PMC9488767

[36] Singh MV Chapleau MW Harwani SC Abboud FM, 2014. The immune system and hypertension. Immunol Res 59: 243–253. 24847766 10.1007/s12026-014-8548-6 PMC4313884

[37] Barri YM, 2008. Hypertension and kidney disease: A deadly connection. Curr Hypertens Rep 10: 39–45. 18367025 10.1007/s11906-008-0009-y

[38] Gasparis AP Kim PS Dean SM Khilnani NM Labropoulos N, 2020. Diagnostic approach to lower limb edema. Phlebology 35: 650–655. 32631171 10.1177/0268355520938283 PMC7536506

[39] Belyaeva II Subbotina AG Eremenko II Tarasov VV Chubarev VN Schiöth HB Mwinyi J, 2022. Pharmacogenetics in primary headache disorders. Front Pharmacol 12: 820214. 35222013 10.3389/fphar.2021.820214 PMC8866828

[40] Ng AW Teoh SC, 2015. Dengue eye disease. Surv Ophthalmol 60: 106–114. 25223497 10.1016/j.survophthal.2014.07.003

[41] Rathakrishnan A Wang SM Hu Y Khan AM Ponnampalavanar S Lum LCS Manikam R Sekaran SD, 2012. Cytokine expression profile of dengue patients at different phases of illness. PLoS One 7: e52215. 23284941 10.1371/journal.pone.0052215 PMC3527385

[42] Kavelaars A Kuis W Knook L Sinnema G Heijnen CJ, 2000. Disturbed neuroendocrine-immune interactions in chronic fatigue syndrome. J Clin Endocrinol Metab 85: 692–696. 10690878 10.1210/jcem.85.2.6379

[43] Wu D Zhang X Deng A Zhang H Zhang Y Tan Q Peng Z Li J Song T, 2020. Dengue fever outbreaks caused by varied serotype dengue virus – Guangdong Province, China, 2019. China CDC Wkly 2: 740–743. 34594750 10.46234/ccdcw2020.137 PMC8422221

[44] Luo S Cui W Li C Ling F Fu T Liu Q Ren J Sun J, 2018. Seroprevalence of dengue IgG antibodies in symptomatic and asymptomatic individuals three years after an outbreak in Zhejiang Province, China. BMC Infect Dis 18: 92. 29471783 10.1186/s12879-018-3000-5 PMC5824482

